# One and five-year efficacy of tension-free vaginal tape (TVT) abbrevo and TVT-obturator in the treatment of stress urinary incontinence: a retrospective study

**DOI:** 10.1186/s12893-024-02446-8

**Published:** 2024-05-11

**Authors:** Jie Hui Wang, Lai Lai Fan, Ying He Chen, Yi jun Wang

**Affiliations:** 1 Department of nursing, The Second Affiliated Hospital, Yuying Children’s Hospital of Wenzhou Medical University, Wenzhou, China; 2 Department of urology, The Second Affiliated Hospital, Yuying Children’s Hospital of Wenzhou Medical University, Wenzhou, China

**Keywords:** Stress urinary incontinence, Tension-free vaginal tape, Abbrevo, Obturator

## Abstract

**Background:**

Surgical interventions are more effective than nonsurgical approaches in providing a cure for stress urinary incontinence (SUI). In this study, we aimed to assess the benefits of tension-free vaginal tape (TVT) abbrevo by comparing its efficacy and complications to those of TVT obturator.

**Methods and results:**

49 and 47 patients at The Second Affiliated Hospital and Yuying Children’s Hospital of Wenzhou Medical University between January 2013 and December 2016 were included in the TVT-O and TVT-A groups, respectively. We evaluate the success rate and perioperative complications associated with TVT-O and TVT-A. A questionnaire that utilized the Patient Global Impression of Improvement (PGI-I) Scale was employed to assess the impact of surgery. Patients were followed up at 1 year, and 5 years after surgery. There were no statistically significant differences found in the efficacy of the TVT-A group and TVT-O group during both the one-year (*p* = 0.4) and five-year (*p* = 0.32) follow-up periods. In the period of one-year follow-up, 95.9% (*n* = 47) of patients in the TVT-O group and 95.8% (*n* = 45) of patients in the TVT-A group demonstrated improvement. During the period of five-year follow-up, 87.8% (*n* = 43) of patients in the TVT-O group and 93.6% (*n* = 44) of patients in the TVT-A group demonstrated improvement.

**Conclusions:**

Based on our findings, TVT-A and TVT-O procedures exhibited similarly high success rates and low frequencies of complications.

## Introduction

Urinary incontinence (UI) is commonly found among women and can have a significant impact on their quality of life [1]. Studies conducted in various countries have reported a wide range of UI prevalence rates, ranging from 5 to 70%. However, most studies have found that around 25–45% of women experience different types of UI. The incidence of UI tends to increase with age, and among women aged 70 years and above, over 40% of the female population is affected. The most frequent type of UI reported in women is stress urinary incontinence (SUI) [2].

According to the definition by the International Continence Society, female SUI refers to the involuntary leakage of urine during physical exertion, coughing, or sneezing [3]. The first line of treatment typically involves behavioral modifications and pelvic floor muscle training [4].Over the last few decades, mid-urethral sling(MUS) surgery has been demonstrated as the preferred treatment for SUI when conservative therapies have proven ineffective. It is estimated that globally, over 5 million mid-urethral mesh slings have been utilized to date [5]. While surgical procedures are more likely to provide a cure for SUI than nonsurgical approaches, it is important to acknowledge that surgical procedures also carry a potential risk of adverse events [6].

The tension-free vaginal tape (TVT) procedure was originally introduced in 1998, but retropubic TVT procedures were associated with complications such as bladder injury, nerve and blood vessel injury [7]. The obturator nerve originates from the lumbar spinal nerves and extends to the thigh, passing through the obturator foramen. During surgery, the helical passers may traverse the obturator foramen, potentially resulting in injury to the obturator nerve and causing pain in the groin or leg. Subsequently, the “inside-out” trans-obturator vaginal tape (TVT-O) technique was introduced in MUS surgery. While TVT-O has demonstrated low complication rates, it may still be associated with nerve damage that can lead to leg and groin pain [8]. TVT-abbrevo (TVT-A) came out a few years later in TVT-O, use of a shorter (12 cm) polypropylene mesh, which minimizes the length of the mesh passing through the adductor muscles [9]. Despite its shorter length, the mesh is still positioned between the obturator membrane and under the mid-urethra, providing the same tension-free support as with TVT-O. However, the evidence of the efficacy and safety of TVT-A versus TVT-O in Asian populations with extended follow-up periods was limited, we conducted the study to assess the efficacy and safety of TVT-A and TVT-O.

## Materials and methods

This study retrospectively analyzed a total of 103 patients who had underwent either TVT-O or TVT-A surgery at The Second Affiliated Hospital and Yuying Children’s Hospital of Wenzhou Medical University between January 2013 and December 2016. Due to the expiration of the surgical material contract, data beyond 2016 was not available. The Ethics Committee of the Second Affiliated Hospital of Wenzhou Medical University (LCKY2020-286) granted approval for this retrospective observational study, due to the nature of this article, the requirement for obtaining informed consent was waived by the board. This study collected various clinical characteristics of the patients, including their age, body mass index (BMI), disease duration, menstrual status, mode of delivery (vaginal or caesarean section), presence of chronic diseases, history of hysterectomy and time of Foley catheter removal after surgery. The chronic diseases considered in this study included diabetes, hypertension, and lung diseases. BMI > 25 is defined as being obesity in Asian population [10].

SUI was defined as the involuntary leakage of urine during physical activities, coughing, sneezing, or exertion. Each patient underwent a comprehensive evaluation, which included a detailed medical history assessment, a clinical examination focused on urogynecological issues, urodynamic studies (UDS) to evaluate bladder function, and tests for urinalysis and urine culture. The UDS were conducted in accordance with the standards of the International Continence Society (ICS). Patients with a history of pelvic malignancy or radiation treatments, urogenital prolapse > stage 1, current or planned pregnancy, prior anti-incontinence surgery, intrinsic urethral sphincter deficiency (ISD), neuromuscular disorders, mixed urinary incontinence, or positive urine culture results for bacterial infection were excluded from the study.

The primary outcome of this study was to determine the success rate of TVT-O and TVT-A procedures. The patients were categorized into three groups based on their responses to a questionnaire that utilized the Patient Global Impression of Improvement (PGI-I) Scale [11]: cure (no SUI episodes), improvement (improved, but still had one or more SUI episodes within 6 months), and unchanged (same or more SUI symptoms as preoperatively, or recurrence). cure and improvement were considered successful [12]. Patients who reported persistent or recurrent symptoms were evaluated to exclude urgency urinary incontinence based on their clinical symptoms and voiding diary. In this study, the TVT-O system (Gynecare; Ethicon) and the TVT-A system (Gynecare; Ethicon) was utilized for the surgical procedures, while all other aspects of the surgery were same. The surgical procedures were carried out by one experienced surgeon.

The secondary outcomes of the study were the short-term and long-term complications associated with TVT-O and TVT-A. Perioperative variables, such as bladder injury, fever, vulvar hematoma, leg or groin pain, dysuria, were recorded. Patients were followed up at 1 year, and 5 years after surgery. Long-term complications related to the surgery, such as chronic pain or mesh exposure, were also recorded.

The data were analyzed with the Statistical Package for Social Sciences (SPSS, IBM, Armonk, NY) version 23.0. Continuous variables were reported as either mean ± SD or medians and interquartile range (IQR). Categorical data were presented as frequencies and percentages. To compare continuous variables, either an unpaired t-test or Mann Whitney-U test was utilized, while the χ2 test or Mann Whitney-U test was used to compare categorical variables, depending on appropriateness. Furthermore, Mann-Whitney test was employed to compare rank variables. A p value < 0.05 was considered statistically significant.

## Result

A total of 103 patients who underwent TVT-O or TVT-A during the study period were primarily included. Among them, 3 patients had a history of pelvic malignancy surgery, 2 patients had undergone previous anti-incontinence surgery, and 2 patients were lost to follow-up at the 5-year mark. In total, 7 patients were excluded from the analysis. The remaining 49 and 47 patients were included in the TVT-O and TVT-A groups, respectively.

Table 1 presents the basic characteristics of two groups, the two groups did not differ significantly in age, BMI, terms of disease duration, menstrual status, mode of delivery, chronic disease, history of hysterectomy and time of Foley catheter removal. The TVT-O group had an average patient age of 53.4 ± 9.7 years (ranging from 36 to 76), while the TVT-A group had an average age of 52.6 ± 9.9 years (ranging from 33 to 82). The average BMI was 24.2 ± 2.8 kg/m^2^ in the TVT-O group and 23.9 ± 2.2 kg/m^2^ in the TVT-A group.

**Table 1:** Baseline characteristics of the study population

Variable	TVT-O (*n* = 49)	TVT-A (*n* = 47)	*P*-value
Age, year	53.4 ± 9.7	52.6 ± 9.9	0.722
BMI, kg/m2	24.2 ± 2.8	23.9 ± 2.2	0.473
Diabetes, n (%)	9 (18.4)	4 (8.5)	0.160
Hypertension, n (%)	14 (28.6)	14 (29.8)	0.896
Lung disease, n (%)	1 (2)	0	0.327
Disease duration, year	7 (3–10)	5 (3–10)	0.349
Menopausal, n (%)	27 (55.1)	20 (42.6)	0.219
Cesarean delivery, n (%)	2 (4.1)	0	0.164
Number of deliveries, n	2 (2–3)	2 (2–3)	0.127
Hysterectomy, n (%)	4 (8.2)	8 (17)	0.192
Catheter removal, day	1 (1-1.5)	1 (1–1)	0.347

BMI: body mass index

Continuous variables were presented as either mean ± SD or medians and interquartile range (IQR). Categorical data were presented as frequencies and percentages (n, %)

There were no statistically significant differences found in the efficacy of the TVT-A group and TVT-O group during both the one-year (*p* = 0.4) and five-year (*p* = 0.32) follow-up periods (Table 2). In the one-year follow-up, 95.9% (*n* = 47) of patients in the TVT-O group and 95.8% (*n* = 45) of patients in the TVT-A group demonstrated improvement or cure, two patients did not experience improvement in the TVT-O group, while two patients in the TVT-A group also did not experience improvement. During the five-year follow-up, 87.8% (*n* = 43) of patients in the TVT-O group and 93.6% (*n* = 44) of patients in the TVT-A group demonstrated improvement, which represents a potential decrease compared to the one-year follow-up period. Specifically, four additional patients in the TVT-O group reported a significant decrease in effectiveness during the five-year follow-up, while one additional patient in the TVT-A group reported the same. During the five-year follow-up, two patients in the TVT-O group who experienced treatment failure during the one-year follow-up underwent a second operation at another hospital and reported improvement. It is worth noting that one additional patient in the TVT-O group during the five-year follow-up experienced treatment failure had a significant increase in weight.

**Table 2:** Effect of TVT-O and TVT-A at one-year and five-year follow-up

	One-year			Five-year		
Effect	TVT-O	TVT-A	P-value	TVT-O	TVT-A	P-value
Cure, n (%)	42 (85.7)	43 (91.5)	0.400	38 (77.6)	41 (87.2)	0.213
Improved, n (%)	5 (10.2)	2 (4.3)		5 (10.2)	3 (6.4)	
Unchanged n (%)	2 (4.1)	2 (4.3)		6 (12.2)	3 (6.4)	

After surgery, three patients (6.1%) in the TVT-O group and two patients (4.3%) in the TVT-A group reported experiencing leg pain (*p* = 0.682). There were no instances of groin pain observed in either group. Leg pain of five patients were resolved within three weeks with COX-2 inhibition analgesic therapy. We evaluated the patient’s voiding status by assessing the postoperative urinary flow rate. In the TVT-O group, four patients (8.2%) experienced dysuria after catheter removal, and three patients (6.4%) in the TVT-A group also reported dysuria (*p* = 0.739). Postoperative urinary restriction in patients is typically caused by bladder outlet obstruction resulting from excessive tightening of the sling. Patients with dysuria received sling mobilization therapy through urethral dilation on both the day of catheter removal and on the third day following the procedure. At the one-week outpatient follow-up, dysuria symptoms in seven patients were resolved. Neither group experienced perioperative complications such as bladder injury, vulva hematoma, fever, or mesh exposure (Table 3).

**Table 3:** Complications associated with TVT-O and TVT-A

Variable	TVT-O (*n* = 49)	TVT-A (*n* = 47)	*P*-value
Leg pain	3 (6.1%)	2 (4.3%)	0.682
Dysuria	4 (8.2%)	3 (6.4%)	0.739
Bladder injury	0	0	ns
Vulva hematoma	0	0	ns
Fever	0	0	ns
Mesh exposure	0	0	ns

ns: not significant

To further explore the effectiveness between the two surgeries, we performed subgroup analyses to determine success rates based on age, BMI, diabetes, hypertension, disease duration, menstrual status, mode of delivery, number of deliveries, and history of hysterectomy (Table 4). In both the one-year and five-year follow-up periods, there was no statistically significant difference observed between the groups of elderly patients, obese patients, diabetic patients, hypertensive patients, patients with long disease duration, patients who delivered via cesarean section, patients with multiple deliveries, and patients with a history of hysterectomy. It is noteworthy that our study uncovered a potential higher success rate among premenopausal patients in the TVT-A group compared to the TVT-O group during the five-year follow-up (*p* = 0.05). However, as the p value did not reach statistical significance, larger sample sizes may be necessary to confirm this finding.

**Table 4:** Subgroup comparisons of success rates at one-year and five-year follow-up

	One-year			Five-year		
Subgroup	TVT-O	TVT-A	P-value	TVT-O	TVT-A	P-value
Age						
≤ 60	36 (97.3)	38 (97.0)	0.97	34 (91.9)	38 (97.4)	0.283
> 60	11 (91.6)	7 (87.5)	0.767	9 (75.0)	6 (75.0)	1
BMI						
≤ 25	30 (96.8)	28 (93.3)	0.538	28 (90.3)	27 (90.0)	0.967
> 25	17 (94.4)	17 (100.0)	0.331	15 (83.3)	17 (100.0)	0.083
Diabetes						
Yes	9 (100.0)	4 (100.0)	1	9 (100.0)	4 (100.0)	1
No	38 (95.0)	41 (95.3)	0.941	34 (85.0)	40 (93.0)	0.243
Hypertension						
Yes	14 (100.0)	13 (92.9)	0.769	12 (85.7)	12 (85.7)	1
No	33 (94.3)	32 (97.0)	0.593	31 (88.6)	32 (97.0)	0.188
Disease duration						
≤ 5	18 (94.7)	25 (96.2)	0.822	18 (94.7)	24 (92.3)	0.750
> 5	29 (96.7)	20 (95.2)	0.798	25 (83.3)	20 (95.2)	0.198
Menopausal						
Yes	26 (96.3)	18 (90.0)	0.388	24 (88.9)	17 (85.0)	0.696
No	21 (95.5)	27 (100.0)	0.268	19 (86.4)	27 (100.0)	0.050
Cesarean delivery						
Yes	2 (100.0)	0 (0)	ns	1 (50.0)	0 (0)	ns
No	45 (95.7)	45(95.7)	1	42 (89.4)	44 (93.6)	0.462
Number of deliveries						
≤ 3	39 (95.1)	43 (95.6)	0.924	36 (87.9)	42 (93.3)	0.381
> 3	8 (100.0)	2 (100.0)	1	7 (87.5)	2 (100.0)	0.617
Hysterectomy						
Yes	4 (100.0)	8 (100.0)	1	4 (100.0)	8 (100.0)	1
No	43 (95.6)	37 (94.9)	0.884	39 (86.7)	36 (92.3)	0.407

ns: not significant

## Discussion

In our research, we found that both TVT-A and TVT-O surgeries exhibited similarly high success rates, with no statistically significant differences in complications. However, it is worth noting that there is limited research available on the comparison between TVT-A and TVT-O. We were only able to find three studies in PubMed that examined this specific comparison, but they had relatively short follow-up durations, one in Italy [13], one in France [14] and one in Korea [12]. Despite their small sample sizes, these studies reported similar success rates.

The primary distinction between the TVT-O and TVT-A procedures is the length of the mesh utilized. This shorter mesh avoids the perforation of the obturator membrane with a scissor and guide, reducing the depth of lateral dissection and lowering the risk of associated neuro-muscular injuries. These adjustments are intended to lower the incidence of postoperative groin and leg pain. However, our research did not identify any benefits of using a shorter mesh. Study reported by Zullo et al. [13] found that the TVT-O procedure resulted in a higher incidence of postoperative groin pain compared to TVT-A (11% vs. 1%). Canel et al. reported that there was less immediate postoperative pain with TVT-A [14]. Patients who reported pain in our study, as well as in the study by Zullo et al., were able to experience resolution of their symptoms in a relatively short period of time. One study suggested that structural weakness leading to uterosacral dislocation, rather than the mesh itself, may be the cause of late pain [15]. In cases where patients experienced persistent pain, the removal of the MUS may be considered as a potential means of resolution or improvement [16].

In our study, seven patients experienced dysuria following catheter removal. Bladder outlet obstruction (BOO) is a common complication of MUS, occurring in 3 to 10% of patients [17]. However, postoperative dysuria may be attributable to other factors related to the surgery, such as anaesthetic drugs, pain, and postoperative swelling. Dysuria resulting from MUS can be resolved through various interventions, including intermittent catheterization, sling mobilization, and sling transection. Early sling mobilization has been shown to be more effective [17, 18]. The way of sling mobilization reported by Moksnes et al. and Pinsard et al. was a traumatic way by pulling the sling gently down between the urethral wall and the suburethral portion of the sling. However, in our study, patients experiencing dysuria received sling mobilization therapy through urethral dilation, which also yielded satisfactory results. In cases where urethral dilation was ineffective, a traumatic approach was preferred.

Our study uncovered a potential higher success rate among premenopausal patients in the TVT-A group compared to the TVT-O group during the five-year follow-up. The premenopausal population tended to have lower ages, lower BMIs, and fewer underlying medical conditions. As reported previously, MUS surgery is generally considered safe for both young and aging patients [19], with significant improvements in outcomes observed. However, it is worth noting that the cure rates tend to decrease with age [20–22]. The common pathophysiologic link between obesity and SUI is an increase in intra-abdominal pressure [23]. And increased BMI has been associated with poorer outcomes following MUS [24–26]. As one patient of our study in the TVT-O group experienced treatment recurrence had a significant increase in weight. And One research reported that concomitant vaginal hysterectomy was associated with a higher risk for failure in TVT-O [27]. For people who underwent MUS, there was few difference between diabetes and no diabetes [28], between patients who had pregnancy or delivery after surgery and those who had not [29], as well as between patients who had undergone hysterectomy and those who had not [30]. It is possible that the combined effects of various factors influenced the efficacy of the MUS procedures. In premenopausal population, the impact of TVT-A may be more significant. However, as the p value did not reach statistical significance, larger sample sizes may be necessary to confirm this finding.

Our study has several limitations that should be acknowledged. Firstly, the research was conducted retrospectively, which may have introduced some biases. Secondly, the analysis of objective cure results was not comprehensive enough to allow for a detailed analysis. Despite these limitations, our study was conducted with a long enough follow-up period to provide meaningful insights into the efficacy and safety.

## Conclusion

Based on our findings, TVT-A and TVT-O procedures exhibited similarly high success rates and low frequencies of complications.

## Data Availability

The data set generated during the current study are available upon request. Data requests can be made via email to the corresponding author.
